# Case Report: An MRI Traumatic Brain Injury Longitudinal Case Study at 7 Tesla: Pre- and Post-injury Structural Network and Volumetric Reorganization and Recovery

**DOI:** 10.3389/fneur.2021.631330

**Published:** 2021-05-17

**Authors:** Stephanie S. G. Brown, Kristen Dams-O'Connor, Eric Watson, Priti Balchandani, Rebecca E. Feldman

**Affiliations:** ^1^Cambridge Intellectual and Developmental Disabilities Research Group, Department of Psychiatry, University of Cambridge, Cambridge, United Kingdom; ^2^Department of Rehabilitation and Human Performance, Brain Injury Research Center, Icahn School of Medicine at Mount Sinai, New York, NY, United States; ^3^Department of Neurology, Icahn School of Medicine at Mount Sinai, New York, NY, United States; ^4^Translational and Molecular Imaging Institute, Icahn School of Medicine at Mount Sinai, New York, NY, United States; ^5^Department of Computer Science, Mathematics, Physics, and Statistics University of British Columbia, Kelowna, BC, Canada

**Keywords:** 7T MRI, diffusion MRI, traumatic brain injury, structural connectivity, case study

## Abstract

**Importance:** A significant limitation of many neuroimaging studies examining mild traumatic brain injury (mTBI) is the unavailability of pre-injury data.

**Objective:** We therefore aimed to utilize pre-injury ultra-high field brain MRI and compare a collection of neuroimaging metrics pre- and post-injury to determine mTBI related changes and evaluate the enhanced sensitivity of high-resolution MRI.

**Design:** In the present case study, we leveraged multi-modal 7 Tesla MRI data acquired at two timepoints prior to mTBI (23 and 12 months prior to injury), and at two timepoints post-injury (2 weeks and 8 months after injury) to examine how a right parietal bone impact affects gross brain structure, subcortical volumetrics, microstructural order, and connectivity.

**Setting:** This research was carried out as a case investigation at a single primary care site.

**Participants:** The case participant was a 38-year-old female selected for inclusion based on a mTBI where a right parietal impact was sustained.

**Main outcomes:** The main outcome measurements of this investigation were high spatial resolution structural brain metrics including volumetric assessment and connection density of the white matter connectome.

**Results:** At the first scan timepoint post-injury, the cortical gray matter and cerebral white matter in both hemispheres appeared to be volumetrically reduced compared to the pre-injury and subsequent post-injury scans. Connectomes produced from whole-brain diffusion-weighted probabilistic tractography showed a widespread decrease in connectivity after trauma when comparing mean post-injury and mean pre-injury connection densities. Findings of reduced fractional anisotropy in the cerebral white matter of both hemispheres at post-injury time point 1 supports reduced connection density at a microstructural level. Trauma-related alterations to whole-brain connection density were markedly reduced at the final scan timepoint, consistent with symptom resolution.

**Conclusions and Relevance:** This case study investigates the structural effects of traumatic brain injury for the first time using pre-injury and post-injury 7 Tesla MRI longitudinal data. We report findings of initial volumetric changes, decreased structural connectivity and reduced microstructural order that appear to return to baseline 8 months post-injury, demonstrating in-depth metrics of physiological recovery. Default mode, salience, occipital, and executive function network alterations reflect patient-reported hypersomnolence, reduced cognitive processing speed and dizziness.

## Introduction

Traumatic brain injury (TBI) is a leading cause of disability worldwide, particularly in young and military populations, with well-documented links to psychiatric and neurodegenerative pathology (1). Patients who experience mild traumatic brain injury (mTBI) typically report vestibular, sensory, cognitive or emotional symptoms that persist for several months after injury (2). Patients experiencing mTBI typically show low frequency of positive MRI findings at 6 months post-injury, highlighting the need for more sophisticated and sensitive imaging techniques in the clinical investigation of mTBI (3, 4). Previous studies have reported the utility of diffusion-weighted imaging and tractography methodology in determining the presence of neuronal injury in cases where conventional neuroimaging findings are negative (5, 6), as they allow examination of fiber- and tract-related pathology. Tracking of the spinothalamic tract in a mTBI case demonstrated thinning and discontinuation of fibers at the subcortical white matter in mTBI patients with no conventional radiological abnormalities (6), and the corticobulbar tract and fornix exhibited similar narrowing and discontinuations in an mTBI case study caused by violence (5). It is probable that white matter damage, common in TBI due to both indirect shearing forces and direct damage, may be a pertinent but often undetected pathophysiology in this population (7). Moreover, it is generally appreciated that injury to the brain resulting from trauma often arises globally, as axons crossing areas of differing tissue density react differently to the mechanical force of the trauma (8). This can cause widespread damage, which may be explored using network and connectivity analyses that draw directly upon anatomically accurate estimations of white matter connection density. A connectomic network approach allows data integration of distinct regions of brain anatomy and connection strength, which makes it a useful methodology for examining both localized and global effects of mTBI on white matter (9).

In this case study, unique due to the rare availability of pre- and post-injury 7 Tesla high-resolution data, we investigated the trajectory of structural changes attributed to mTBI. The multiple time-point pre-injury data is an uncommon strength to the present research, as longitudinal data can be examined in both healthy and post-injury settings. Moreover, we aimed to investigate and characterize disparities between conventional structural MRI and diffusion-weighted connectomic findings. In this report, we hope to illustrate how recent developments in the field of computational neuroimaging, such as morphometric subcortical segmentations and network theory, may aid in the identification of suitably sensitive biomarkers of brain injury.

## Methods

### Case Description

A 38-year-old female was involved in a motor vehicle accident in which she was a pedestrian hit by a car turning into the intersection she was crossing. She was thrown across the road where her head hit the curb. She was transported to the nearby hospital where acutely, the patient was dizzy, faint and mildly confused. Head CT revealed subcutaneous soft tissue swelling over the right parietal bone. There was no evidence of acute territorial infarction or intracranial hemorrhage. Ventricles and sulci appeared normal in size and configuration for the patient's age. There was no midline shift or other mass effect, and gray-white matter differentiation was maintained throughout the brain. The patient received surgical staples to close a laceration over the right parietal bone and was discharged home. The patient reported minimal headaches or nausea, but dizziness, daytime fatigue, hypersomnolence, reduced problem-solving skills and slowed cognitive processing persisted for several weeks following the injury. She returned to work the day after the injury, working slightly reduced hours to accommodate fatigue. Full recovery, defined as full symptom resolution and return to baseline function, was achieved ~6 months post-injury. The patient gave fully informed consent for participation in the presented research. Institutional Review Board (IRB) approval for human research was obtained for this experiment from the Program for Protection of Human Subjects at the Icahn School of Medicine at Mount Sinai.

### Longitudinal Data Acquisition

The patient had undergone two scanning sessions at 7 Tesla prior to the head injury as a healthy control. Two more scans were acquired post-injury. The scan times in relation to injury were as follows: 23 months prior to injury, 12 months prior to injury, 2 weeks post-injury, and 8 months post-injury. All MRI scanning was performed using the same Siemens 7T scanner. Clinical assessment and an initial head CT were carried out immediately after injury, and neurocognitive testing was administered by a trained clinician 18 months post-injury.

### Clinical Neurocognitive Data Acquisition

A brief battery of performance-based neurocognitive tests was administered to estimate premorbid intellectual functioning and quantitatively confirm cognitive recovery 18 months post-injury (10).

### MRI Acquisition

Included in each MRI protocol was a T_1_-weighted Magnetization Prepared 2 Rapid Acquisition Gradient Echo (MP2RAGE) (11), a T2-weighted Turbo Spin Echo (TSE), and a diffusion MRI.

The MP2RAGE sequence obtains improved gray-white contrast at high field compared to the classic MPRAGE acquisition (11). High spatial resolution voxel size was 0.8 mm isotropic, TR/TE = 6,000/3.2 ms, TI1(θ_1_)/TI2(θ_2_) = 1,050(5°)/ 3,000(4°) ms and total acquisition time was 7:26 minutes. From the MP2RAGE dataset, a total of four images were reconstructed from (a) data acquired after inversion time (TI) of 1,050 ms, (b) data acquired after TI of 3,000 ms, (c) T1 relaxation maps calculated from (a) and (b), and (d) uniform-denoised (UNIDEN) images calculated from (a) and (b). An in-plane acceleration factor of 3 was used.

Two TSE structural images were obtained at high in-plane resolution (0.4 × 0.4 mm^2^), a slice thickness of 2 mm, TR/TE = 6,900/69 ms and θ = 150°. An in-plane acceleration factor of 2 was used. The first T2-TSE was obtained with a 6:14 min acquisition time in a coronal-oblique orientation where the imaging plane was aligned perpendicular to the long axis of the hippocampus. The second T2-TSE was obtained in an axial orientation; the imaging plane alighted along the axis connecting the anterior commissure and the posterior commissure (AC-PC). The acquisition time for the second T2-TSE scan was 6:50 min.

Diffusion MRI data were collected using a single-shot spin-EPI sequence aligned axially with an isotropic resolution of 1.05 mm, an in-plane acceleration factor of 3, a multi-band acceleration factor of 2 and TR/TE = 6,900/67 ms. The diffusion sequence was a paired acquisition with reversed phase encoding in the AP/PA direction, and each pair had 64 diffusion encoding directions (*b* = 1,200 s/mm^2^) and 4 unweighted scans (*b* = 0 s/mm^2^). Total scan time for the paired acquisition was 20 min.

### Structural MRI Analysis

The FreeSurfer “recon-all” pipeline (version 6.0) (12) was used to carry out the following processing steps on T1-weighted structural data: motion correction, intensity correction, transform to Talairach space, intensity normalization, skull strip, subcortical segmentation, neck remove, subcortical labeling, segmentation statistics, a second intensity correction using brain only (after skull strip), white matter segmentation, subcortical mass creation, brain surface creation, surface inflation, automatic topology fixer, cortical thickness/pial surfaces, cortical ribbon mask, spherical inflation of the brain surface, ipsilateral surface registration, contralateral surface registration, resampling of the average atlas curvature to subject, cortical parcellation, and creation of summary table for parcellation statistics. As the T1-weighted data had a submillimeter isotropic voxel size, the “-hires” flag was used to preserve enhanced spatial resolution (13).

**Figure 1:**
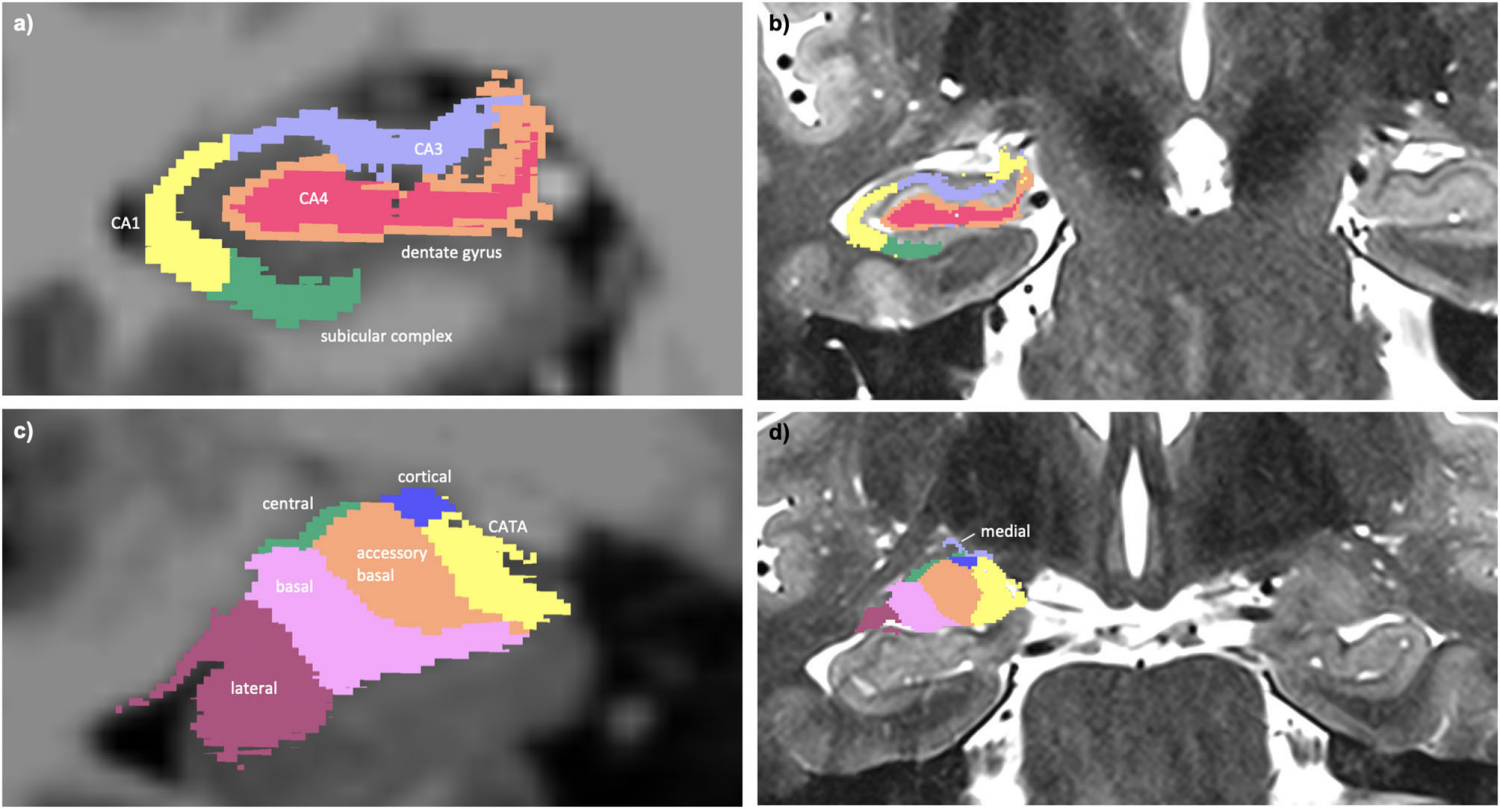
**(a)** Multi-spectral hippocampal subfield segmentation (CA1, CA3, CA4, dentate gyrus, and subicular complex) with underlay of axial T1-weighted data. **(b)** Hippocampal subfield segmentation with underlay of axial T2-weighted data. **(c)** Multi-spectral amygdala subnuclei segmentation (lateral, basal, accessory basal, central, cortical, medial nuclei, and corticoamygdaloid transition area (CATA) with underlay of axial T1-weighted data. **(d)** Amygdala subnuclei segmentation with underlay of axial T2-weighted data.

Hippocampal subfield (14) and amygdala subnuclei segmentation (15) was carried out using FreeSurfer 6.0 development version. A multi-spectral approach was used, utilizing both the T1-weighted and T2-weighted images, leveraging the enhanced resolution of the T2-weighted image to provide additional anatomical information. This subcortical segmentation is visualized in Figure 1.

To investigate test–rest variability, blinded re-runs of the imaging processing were carried out, and pairwise coefficients of variation were calculated per measure by dividing the standard deviation by the mean and multiplying by 100 to produce a percentage.

### Diffusion MRI Analysis

Denoising of the diffusion weighted data was performed using MRTrix two-shell phase-reversed processing (16, 17). Segmented and parcellated structural images from the FreeSurfer “recon-all” pipeline were used for whole brain masking (12). B_1_ field inhomogeneity correction was carried out (18) and the fiber orientation distributions (FODs) were created from the diffusion data using constrained super-resolved spherical deconvolution (19). Estimation of the diffusion tensor was done using iteratively reweighted linear least squares methodology (20). The tensor image was used to create a whole brain map of fractional anisotropy (FA) (21). Mean FA was extracted from cerebral white matter hemispheric masks created by the FreeSurfer pipeline.

Co-registration of anatomical images into diffusion space was then carried out using Statistical Parametric Mapping software (SPM12). Degree of spline interpolation was 4. The MRTrix command “5ttgen” was used to generate a five tissue-type segmentation image, utilizing the FreeSurfer outputs, to use in anatomically constrained tractography (22). A segmented mask image was then created for the seeding of tractography streamlines at the gray-white matter interface (22). The fiber orientation distributions were then used to create whole brain tractograms for each participant (23). Ten million streamlines were generated from the probabilistic tractography per brain. Individual step size for the streamlines was 0.1 mm × voxel size, the fiber orientation distribution amplitude cut-off was 0.05 and the maximum angle between successive steps was 90° × step size × voxel size. Seeds were placed in the gray white matter interface. Spherical deconvolution informed filtering (SIFT2) was applied to the tractograms, the purpose of which was to weight streamlines based on likelihood of anatomical accuracy, remove spurious streamlines from further analysis and ensure data that is highly representative of ground-truth biology (24). A structural connectome, based on node-to-node connection density, was created using MRTrix (25).

Structural connectomes at different timepoints were compared by custom functions that performed elementwise subtraction of the matrices in MATLAB. Similarly, variability of the connectomes was assessed by stacking matrices into a 3-dimensional array and computing the mean and standard deviation along the *z*-axis for each network edge. To investigate variability between scan timepoints, specifically to examine test–retest variation, the two pre-injury connectomes were compared using co-efficients of variation, calculated elementwise for each edge of the connectivity matrix by dividing the standard deviation by the mean and multiplying by 100 to produce a percentage. The mean co-efficient of variation was calculated by averaging the co-efficients of variation across the whole matrix. To determine a streamline threshold of the connectome with an acceptable level of variability, mean matrix co-efficients of variation were calculated for the following streamline thresholds: 25, 50, 100, 200, 400, 800, 1,600, 3,200, 6,400, 12,800, and 25,600. Actual streamline thresholding was subsequently set at 15,000, discarding edges consisting of streamline bundles with less density than the threshold.

## Results

### Clinical Neurocognitive Data

This high-achieving woman with a history of academic excellence throughout 20 years of formal education had an estimated premorbid intellectual ability in the high average-superior range (10). At the time of testing her performance-based intellectual quotient (IQ) was in the superior range, consistent with expectation. She demonstrated a relative strength in verbal comprehension (96th percentile) as compared to perceptual reasoning (>99th percentile). Performance on tests of contextual and non-contextual verbal memory was consistently above the 98th percentile, while visual memory performance was at the 34th percentile (Average range). Tests of complex attention and working memory were generally above the 85th percentile (High Average range), and tests of verbal fluency were variable (semantic fluency 38th percentile; phonemic fluency 96th percentile). Performance on timed tests of sequencing and task-switching were below expectation (<1st percentile – 62nd percentile) while untimed tests of these higher order executive functions were well within expectation (>96th percentile). Overall, performance on neurocognitive tests indicate superior intellectual ability with performance 18 months post-TBI largely consistent with expectations; impaired performance on select timed tests suggest a tendency to sacrifice speed to ensure accuracy which may reflect a compensatory strategy.

### Structural MRI Analysis

The subcortical segmentation of the amygdala nuclei and hippocampal subfields did not reveal any clear changes between the scanning timepoints. The average test-retest coefficient of variation for the hippocampal subfield segmentation was 1.4%, and 5.2% for the amygdala nuclei. Considering this estimation of variability within the image processing, the data did not reveal evidence of volumetric change to the hippocampus or amygdala post-injury, either at a whole or substructure level.

**Figure 2:**
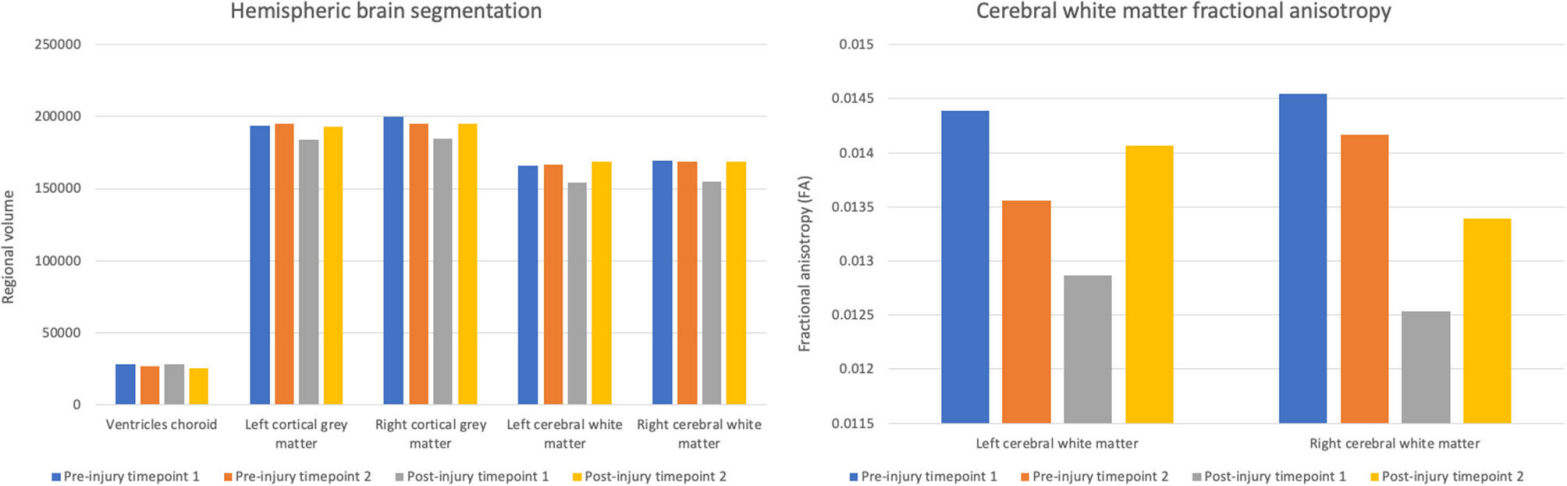
Volumes of choroid ventricles, hemispheric gray matter, and hemispheric white matter and fractional anisotropy of the hemispheric cerebral white matter across scanning timepoints.

At post-injury timepoint 1, the right and left hemispheric brain segmentation revealed lower cortical gray matter and cerebral white matter volume compared to other scanning timepoints. No change was apparent in ventricle volume (Figure 2). The average test-retest variation co-efficient for each of these variables was <0.001%, significantly less than the observed change post-TBI.

### Diffusion MRI Analysis

Concurrent with the changes in the structural volumetrics, FA of the cerebral white matter was markedly reduced in both hemispheres in the first scan following the head trauma. In both the left and right hemispheres, the final timepoint scan revealed a subsequent increase of FA to levels similar to those pre-injury (Figure 2).

**Figure 3:**
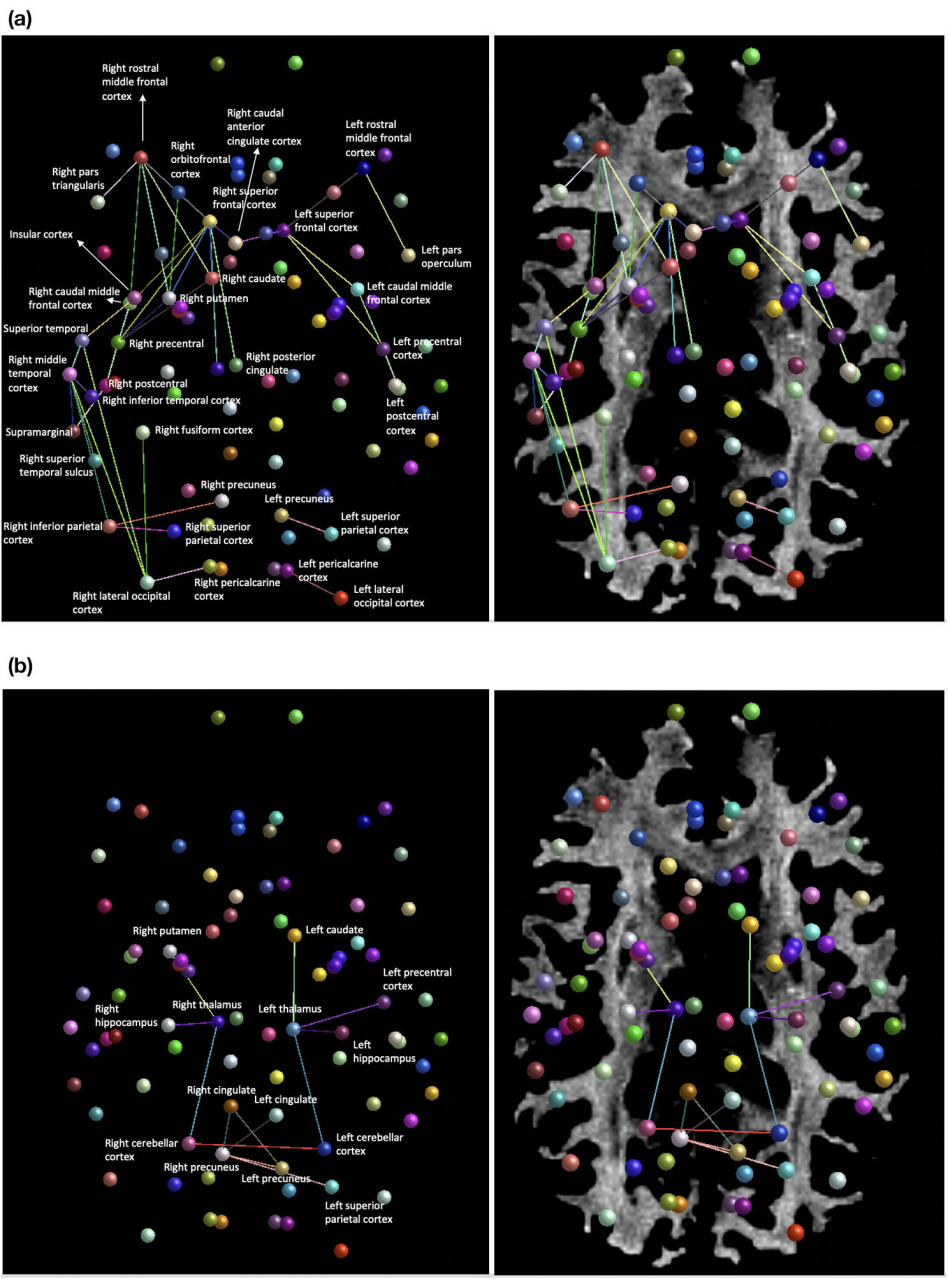
**(a)** Areas of decreased connection density of the structural network mean post-injury compared to mean pre-injury. **(b)** Regions of increased connection density of the structural network mean post-injury compared to mean-pre-injury.

Averaging of the structural diffusion MRI connectomes pre- and post-injury revealed a widespread decrease in connectivity after the patient's head trauma, mainly involving connections between cortical regions (Figure 3A). To a lesser degree, mean pre- to post-injury comparison also revealed some increased connectivity, primarily in subcortical areas and the forebrain (Figure 3B). A comparison between the first and second post-injury connectome matrices was then carried out, to investigate if changes to the patient's structural connectivity post-TBI were consistent over time. The results showed that at post-injury timepoint 1, connection density was extensively reduced, but this decrease in connectivity was partially reduced by post-injury timepoint 2.

## Discussion

Structural high-resolution neuroimaging data in this mTBI case study revealed reduced cerebral white matter and cortical gray matter volumes post-injury that appeared to restore to pre-injury quantifications by the 8-month post-TBI MRI acquisition. Whole brain white matter fractional anisotropy demonstrated a concurrent pattern of change, with marked short-term reductions post-injury returning to a baseline level by the 8-month following TBI. The structural connectome, derived from tractography-based connection density metrics, showed that post-injury connectivity was reduced extensively between cortical nodes, in particular in the right parietal injury site. To a lesser degree, limbic, and forebrain regions were intra-hyperconnected. The final post-injury scanning timepoint showed globalized increases in connection density compared to the primary post-injury data, suggestive of recovery of the network. We highlight here the rare availability of pre-injury data, which gives significant benefits to interpretability compared to post-injury only research into mTBI.

Interestingly, subcortical segmentation and analysis of regional brain volumetrics did not reveal changes post-TBI, indicating a robustness of the limbic structures in this case. This is in contrast to previous reports of the hippocampal and amygdala structures being promising predictors of outcome when analyzed at a gross level (26); however, severity and type of TBI are significant contributors to heterogeneity. Our results suggest that subcortical volumetrics may not be a sensitive measure of mTBI pathology in all cases. At a whole brain level, it appeared that quantification of hemispheric white and gray matter volumes was a more effective metric of brain changes post-TBI in this case, especially when considering the minimal test–retest variability.

The mechanical properties of the white matter make it particularly vulnerable to injury in TBI (5), which was a prominent motivation for the use of high spatial resolution diffusion-weighted MRI in this case investigation. Our findings show that primary post-injury connectivity is reduced in a widespread manner, mainly between cortical nodes. Similarly, a study of mTBI patients and matched controls revealed decreased fractional anisotropy in the association, commissural and projection white matter tracts, indicative of reduced connectivity, which partially resolved 6 months post-injury (27). In addition, our results identified increased white matter connectivity in the limbic and forebrain regions post-injury compared to pre-injury data. Resting-state investigation into mTBI has shown comparable hyperconnectivity in the limbic system post-injury (28), and thalamic circuitry, in particular, has been shown to be a key underlying factor in mTBI recovery (29). Alterations of the connectome post-mTBI in the present case were further substantiated by widespread corroborative changes in fractional anisotropy, suggesting that after injury, white matter microstructure was changed in a way highly indicative of axonal damage (30).

The involvement of the posterior cingulate, precuneus and prefrontal cortices in the decline of structural network density implicates decreased cohesiveness of the default mode system. Decreased functional coupling of the default mode network, in particular in the frontal regions, has been shown to occur during sleep in both humans and primates, suggesting that default mode cohesiveness may be required to maintain conscious states (31). The default mode white matter damage detected in the present study; therefore, may be a contributory factor in the patient's reported hypersomnolence and fatigue post-injury. Similarly, the superior frontal and orbitofrontal regions are integral sites for executive processing (32), and the observed decreases in network connection density here tally closely with the patient's symptomology of delayed cognitive processing post-injury. Decreased white matter connectivity of the insular cortex implicates decreased salience network integration, which may be another possible physiological correlate of cognitive slowing via diminished attentional regulation (33). Alterations to the thalamic-occipital lobe circuitry, which forms the posterior portion of the primary visual pathway (34), may also underlie symptomatic dizziness post-TBI. Localized increases in the mean connection density of the thalamus post-injury, a factor previously reported as a protective feature against long-term pathological effects in mTBI (29), is also seen in this patient, and may reflect compensatory plasticity promoting sensory relay and upstream network integration (35).

Additionally, consistent with previous studies of diffusion-weighted imaging in TBI (27), the current data show recovery of the brain white matter, which is consistent with symptomatic recovery per subjective report at ~6 months post-injury. However, the structural network at post-injury timepoint 2 exhibits some evidence for enduring TBI-related change, given that compared to pre-injury data, the final timepoint connectivity shows some disorganization, albeit markedly improved compared to the initial post-injury network. The functional implications of long-term reorganization of the network appear to be minimal, although may account for the few isolated areas of cognitive performance that were lower than expected in patient at 18 months post-injury. Taken together, our findings show that diffusion MRI connectomics and microstructural measurements may be sensitive to clinical status.

This 7 Tesla case report demonstrates novel evidence of widespread connectivity and microstructural changes at a highly granular level after mTBI, where conventional neuroimaging at a clinical level showed no radiological abnormalities. Moreover, we demonstrate the disparity between T1- and T2-weighted acquisition-derived information and diffusion MRI and suggest that diffusion-weighted investigation of TBI symptomology may be of significant use in clinical practice.

## Data Availability Statement

The raw data supporting the conclusions of this article will be made available by the authors, without undue reservation.

## Ethics Statement

The studies involving human participants were reviewed and approved by Regional ethics committee, Icahn School of Medicine at Mount Sinai. The patients/participants provided their written informed consent to participate in this study.

## Author Contributions

SB, KD-O'C, PB, and RF contributed substantially to the study conception and design, drafted, and revised the article for important intellectual content and gave final approval of the version to be published. EW provided guidance for cognitive assessment. SB carried out all data processing and neuroimage analyses. All authors contributed to the article and approved the submitted version.

## Conflict of Interest

The authors declare that the research was conducted in the absence of any commercial or financial relationships that could be construed as a potential conflict of interest.
